# Supplementary material for: Lack of Sex Disparity in Oral Anticoagulation in Atrial Fibrillation Patients Presenting with Ischemic Stroke in a Rural Population
Source: J Clin Med. 2021 Oct 12;10(20):4670. doi: 10.3390/jcm10204670 (PMC8537827; doi:10.3390/jcm10204670)
Supplement: Supplementary file 1 [file jcm-10-04670-s001.zip › jcm-1377367-SI.pdf]

Supplemental Material

Supplemental Result R1. Risk stratification by $CHADS_2$ score

When using $CHADS_2$ to stratify the risk of stroke in AF patients, 866 (81.5%) patients had $CHADS_2 \geq 2$. Undertreatment was found in 438 (50.6%) patients (Supplemental Table S1). Of these 866 patients, 465 were women and 401 were men. Undertreatment was not found to be significantly different between the two [women: 244 (52.5%) vs. men: 194 (48.4%), p = 0.257] nor was male sex a significant factor in multiple logistic regression. Undertreatment rate was not significantly different even after dividing the patients into four age groups based on age quartiles, and male sex was not a significant factor in multiple logistic regression in any of the sub-groups (Supplemental Table S1).

Supplemental Table S1. Anticoagulant undertreatment rates and logistic regression for patients with a $CHADS_2$ score ≥ 2 stratified

		Undertreatment Rates				Multiple Logistic Regression model for Undertreatment	
**Group**		**All patients**	**Female patients**	**Male patients**	**p-value (Female vs Male)**	**Male Sex OR [95% CI]**	**p-value**
**Total**	**N**	866	465	401			
	**Undertreated**	438 (50.6%)	244 (52.5%)	194 (48.4%)	0.257	0.82 [0.6 – 1.12]	0.21
**Group 1 (44.4 – 74.9 years)**	**N**	217	86	131			
	**Undertreated**	108 (49.8%)	45 (52.3%)	63 (48.1%)	0.637	0.75 [0.4 – 1.41]	0.37
**Group 2 (74.9 – 81.5 years)**	**N**	217	97	120			
	**Undertreated**	98 (45.2%)	49 (50.5%)	49 (40.8%)	0.198	0.72 [0.38 – 1.35]	0.31
**Group 3 (81.5 – 87.3 years)**	**N**	216	120	96			
	**Undertreated**	106 (49.1%)	56 (46.7%)	50 (52.1%)	0.513	1.14 [0.62 – 2.11]	0.67
**Group 4 (87.3 – 89.0 years)**	**N**	216	162	54			
	**Undertreated**	126 (58.3%)	94 (58.0%)	32 (59.3%)	1.0	0.92 [0.42 – 2.05]	0.83

Supplemental Result R2. Risk of bleeding using the *limited HAS-BLED*

Among the 1015 patients, the median score for the *limited HAS-BLED* was not significantly different between the two sexes (women: median 2 [IQR 1 – 2], men: median 2 [IQR 1 – 3], p = 0.109). *Limited HAS-BLED* ≥ 3 was seen more in men [130 (24.3%) female patients vs. 141 (30.4%) male patients, p = 0.035]. After inclusion of *limited HAS-BLED* ≥ 3 to the multiple logistic regression model, the association of male sex with undertreatment was still not found to be significant (OR 0.82, CI 0.61 – 1.09, p = 0.18).

Supplemental Table S2. Summary of Relevant Studies

Author, Year, and Region	Sample (n)	Type of Study	Results	Findings
Essien et al.<br>2020<br>United States	Total Patients:<br>42,952<br>Men: 25,017 (58.2%)<br>Women: 17,935 (41.8%)	Retrospective study	Women less likely to initiate OAC (OR 0.59, CI 95% 0.55-0.64)	Among a group of Medicare beneficiaries, women less likely to initiate OACs
Thompson et al.<br>2017<br>United States	Total Patients:<br>691,906<br>Men: 356,150 (51.5%)<br>Women: 335,756 (48.5%)	Retrospective study	Undertreatment of women at all CHA_2DS_2-VASc score<br>$p < 0.001$	Women had a higher risk of undertreatment across all levels of CHA_2DS_2-VASc
Yong et al.<br>2020<br>United States	Total Patients:<br>358,649<br>Men: 205,756 (57%)<br>Women: 152,893 (43%)	Retrospective study	**Undertreatment**<br>CHA_2DS_2-VASc ≥ 2<br>49.5% Female vs. 42.9% Male<br>$p < 0.0001$	Among patients with CHA_2DS_2-VASc ≥ 2, women more likely to be undertreated
Bhave et al.<br>2015<br>United States	Total Patients:<br>517,941<br>Men: 209,788 (41%)<br>Women: 308,153 (59%)	Retrospective study	**Treatment**<br>35.0% Female vs. 38.8% Male<br>**OAC Odds ratio**<br>OR [95% CI]<br>0.86 [0.86-0.87]<br>$p <.001$	Women are at higher risk of undertreatment with OAC
Xiong et al.<br>2015<br>China	Total Patients:<br>2,442<br>Men: 1,376 (56.3%)<br>Women: 1,066 (43.7%)	Retrospective study	OAC use during hospitalization was 33.3% without sex differences	No sex disparity in OAC treatment
Li et al.<br>2019<br>China	Total Patients:<br>14,723<br>Men: 9,078 (61.7%)<br>Women: 5,645 (38.3%)	Prospective study	**Undertreatment**<br>CHA_2DS_2-VASc ≥ 2<br>**Aged < 75**<br>32.1% Female vs. 30.1% Male<br>$p =0.081$;<br>**Aged ≥ 75**<br>34.6% Female vs. 35.3% Male<br>$p =0.700$	No sex disparities found among males and females with CHA_2DS_2-VASc ≥ 2
Lip et al.<br>2014<br>Europe	Total Patients:<br>3,119<br>Men: 1,859 (59.6%)<br>Women: 1,260 (40.4%)	Prospective study	**Treatment**<br>CHA_2DS_2-VASc ≥ 2<br>95.3% Female vs. 76.2% Male<br>$p < 0.0001$	High rate of OAC prescription among females
Lip et al.<br>2015<br>International	Total Patients:<br>17,184<br>Men: 9,654 (56.2%)<br>Women: 7,530 (43.8%)	Prospective study	**Undertreatment**<br>CHA_2DS_2-VASc ≥ 2<br>38.4% Female vs. 35.4% Male	Rates of anticoagulant usage are similar in men and women.
Mazurek et al.<br>2018<br>International	Total Patients:<br>15,092<br>Men: 8,220 (54.5%)<br>Women: 6,872 (45.5%)	Prospective study	**Treatment**<br>**Globally**<br>CHA_2DS_2-VASc ≥ 2<br>81.4% Female vs. 83.0% Male<br>**North America**<br>75.9% Female vs. 80.4% Male	Rates of anticoagulant usage are similar in men and women, except in North America
Marzona et al.<br>2019<br>Italy	2002<br>Total Patients:<br>35,595<br>Men: 17,689 (49.7%)	Retrospective study	**OAC Odds Ratio**<br>OR [95% CI]<br>**<65 years**<br>2002: 1.51 [1.35-1.69]	Female patients in the ≥ 75 age group had an increased risk of OAC undertreatment

	Women: 17,906 (50.3%)<br>2013<br>Total Patients:<br>33,295<br>Men: 17,323 (52.0%)<br>Women: 15,972 (48.0%)		2013: 1.35[1.17-1.57]<br>**65-74 years**<br>2002: 1.14 (1.06-1.241<br>2013: 1.12[1.02-1.24]<br>**≥75 years**<br>2002: 0.92[0.86-0.981<br>2013: 0.78 [0.73-0.82]	
Loikas et al.<br>2017<br>Sweden	Total Patients:<br>41,702<br>Men: 23,198 (55.6%)<br>Women: 18,504 (44.4%)	Cross-sectional study	**Treatment**<br>**2007-2011**<br>47.8% Female vs.<br>53.2% Male<br>**2011-2015**<br>70.5% Female vs.<br>70.1% Male	Sex differences reported for 2007-2011 with low OAC usage among women, but difference disappeared in 2011-2015 except for females 80 years and older
